# Culture-Independent Identification of Nontuberculous Mycobacteria in Cystic Fibrosis Respiratory Samples

**DOI:** 10.1371/journal.pone.0153876

**Published:** 2016-04-19

**Authors:** Lindsay J. Caverly, Lisa A. Carmody, Sarah-Jane Haig, Nadine Kotlarz, Linda M. Kalikin, Lutgarde Raskin, John J. LiPuma

**Affiliations:** 1 Department of Pediatrics and Communicable Diseases, University of Michigan Medical School, Ann Arbor, Michigan, United States of America; 2 Department of Civil & Environmental Engineering, University of Michigan, Ann Arbor, Michigan, United States of America; University of Alabama-Birmingham, UNITED STATES

## Abstract

Respiratory tract infections with nontuberculous mycobacteria (NTM) are increasing in prevalence and are a significant cause of lung function decline in individuals with cystic fibrosis (CF). NTM have been detected in culture-independent analyses of CF airway microbiota at lower rates than would be expected based on published prevalence data, likely due to poor lysing of the NTM cell wall during DNA extraction. We compared a standard bacterial lysis protocol with a modified method by measuring NTM DNA extraction by qPCR and NTM detection with bacterial 16S rRNA gene sequencing. The modified method improved NTM DNA recovery from spiked CF sputum samples by a mean of 0.53 log_10_ copies/mL for *M*. *abscessus* complex and by a mean of 0.43 log_10_ copies/mL for *M*. *avium* complex as measured by qPCR targeting the *atpE* gene. The modified method also improved DNA sequence based NTM detection in NTM culture-positive CF sputum and bronchoalveolar lavage samples; however, both qPCR and 16S rRNA gene sequencing remained less sensitive than culture for NTM detection. We highlight the limitations of culture-independent identification of NTM from CF respiratory samples, and illustrate how alterations in the bacterial lysis and DNA extraction process can be employed to improve NTM detection with both qPCR and 16S rRNA gene sequencing.

## Introduction

Nontuberculous mycobacterial (NTM) infections affect approximately 13% of individuals with cystic fibrosis (CF) and are increasing in prevalence[1,2]. While NTM infections can result in significant lung function decline and death, the clinical course after NTM acquisition is highly variable[3,4]. Predictors of NTM acquisition and subsequent disease course are largely unknown.

Understanding the relationship(s) between NTM infection and other members of the microbial communities inhabiting the CF airways offers potential insight into NTM disease pathogenesis. Although prior investigations have not consistently identified co-infection with other bacterial pathogens as risk factors for NTM acquisition or disease course[1,5,6], these studies have been limited by the use of culture-based bacterial detection, which focuses on identifying known CF pathogens[7]. Studies employing DNA-sequencing based approaches to characterize CF airway bacterial communities offer an opportunity to assess the impact of a broader range of species on NTM acquisition and/or disease. Culture-independent analyses have, for example, identified differences in the airway microbiomes of non-CF patients with pulmonary *Mycobacterium tuberculosis* (MTb) infection compared to healthy controls[8,9]. Differences in airway microbiota in individuals with pulmonary MTb are also associated with treatment response[10].

We previously sequenced the bacterial 16S rRNA genes in more than a thousand sputum samples from individuals with CF[7,11–14]. Review of these data showed an unexpected lack of DNA sequences classified as *Mycobacterium*, particularly in 16 samples from eight individuals that were culture-positive for NTM. Suspecting that the failure to detect NTM DNA resulted from inadequate lysing of NTM cells during DNA extraction from sputum, we assessed the effect of various alterations to our standard bacterial cell lysis protocol on enhancing NTM cell lysis and developed a modified bacterial cell lysis protocol.

We compared the performance of this modified lysis protocol to our standard protocol with respect to NTM DNA recovery from both NTM-spiked sputum samples and NTM culture-positive respiratory samples. We also assessed the impact of the lysis protocol modifications on 16S rRNA gene sequence based measures of bacterial community structure in the NTM culture-positive respiratory samples.

## Materials and Methods

### Development of modified lysis protocol

Prior efforts by other investigators to enhance NTM cell lysis for DNA extraction have demonstrated that a combination of bead beating and enzymatic extraction results in better NTM cell lysis as compared to either enzymatic or chemical lysis alone[15–18]. In preliminary experiments, we confirmed these findings on CF respiratory samples, as well as confirmed findings that NTM DNA yield did not improve with prolonged duration of enzymatic lysis[19], alternate methods of physical lysis (e.g. freeze-thaw)[18,20], or with extending bead beating times beyond two minutes[19,21]. We observed that the greatest increase in NTM DNA yield from CF respiratory samples was obtained by altering the bead beating conditions of our standard protocol. Specifically, we modified our standard lysis protocol by changing from glass beads to the denser zirconium beads[21] and by decreasing the sample volume during bead-beating.

### Respiratory samples and NTM strains

The sputum and bronchoalveolar lavage (BAL) samples included in this study are part of a large collection of respiratory samples obtained during the course of routine care of individuals with CF followed at the University of Michigan Health System (UMHS) CF care centers from 2006–2015. Sample collection and medical record review were approved by the University of Michigan Institutional Review Board (HUM00048991 and HUM00080378). Waiver of subject informed consent was granted as this study was limited to a retrospective analysis of existing unique-identifier-encoded samples and clinical data. The Electronic Medical Record Search Engine (EMERSE) was used to extract relevant clinical data from the medical record[22]. Sputum and BAL samples were aliquoted and stored neat at -80°C. Prior to DNA extraction, sputum and BAL sample aliquots were thawed on ice then incubated with an equal volume of 10% Sputolysin® at 37°C for 30 min with pulse vortexing every 5 min to achieve homogenization.

All NTM strains used in spiking experiments were stored in Mueller-Hinton liquid media with glycerol at -80°C prior to being grown on Middlebrook 7H11 solid media at 37°C. Bacteria were suspended in sterile phosphate-buffered saline and density was quantified by optical density and confirmed by plating serial dilutions.

### NTM- spiked sputum samples

Sputum samples were identified from an individual with CF who had no history of NTM infection based on annual negative NTM sputum cultures. Sputum samples were pooled and homogenized, then spiked with 10^7^ cfu/ml of one of four NTM strains: *Mycobacterium abscessus* complex (MABSC) ATCC 19977, *Mycobacterium avium* complex (MAC) ATCC 25291, and one clinical isolate each of MABSC and MAC that were obtained from the UMHS clinical microbiology laboratory. Replicate 350 μL aliquots of spiked sputum were processed by the standard or the modified lysis protocols prior to DNA extraction and qPCR as described below. Six paired, spiked sputum samples were tested for each ATCC strain, and four paired, spiked sputum samples were tested for each clinical strain.

### NTM culture-positive samples

Twelve individuals with CF and a history of NTM infection were identified with EMERSE. From these 12 individuals, 15 samples (11 sputum and four BAL) were identified in our collection that were NTM culture-positive (seven MABSC and eight MAC). NTM culture had been performed by the UMHS clinical microbiology laboratory. Smear positivity was defined as the presence of at least one organism per high powered field. NTM species identification was confirmed in each case by the Michigan Department of Community Health. Sample appearance was assessed with respect to consistency and presence/absence of gross blood at the time of DNA extraction. After thawing and homogenization, duplicate aliquots of the NTM culture-positive samples were processed by the standard or the modified lysis protocol prior to DNA extraction, qPCR, and 16S rRNA gene sequencing as described below.

### Bacterial lysis and DNA extraction

#### Standard lysis protocol

A 350 μL aliquot of the homogenized sample was mixed with 0.9 volume of MagNA Pure Bacterial Lysis Buffer (Roche Applied Science, Indianapolis, IN), lysozyme (final concentration, 2.9 mg/mL; Sigma-Aldrich Corp., St. Louis, MO), and lysostaphin (final concentration, 0.14 mg/mL; Sigma-Aldrich), then incubated for 30 min at 37°C[23]. Samples were transferred to a tube containing 0.1 mm glass beads (MoBio Laboratories, Carlsbad, CA) and agitated in a Mini-Beadbeater-9 (Biospec Products Inc., Bartlesville, OK) for 1 min at the maximum setting. Samples were digested with Proteinase K (final concentration, 1 mg/mL) and incubated for 10 min at 65°C, agitated for an additional 1 min in the Mini-Beadbeater-9, and then incubated for an additional 10 min at 95°C. DNA purification was performed using an automated nucleic acid purification platform (MagNa Pure Compact System, Roche) using the manufacturer’s DNA Bacteria v3.1 protocol.

#### Modified lysis protocol

A 350 μL aliquot of the homogenized sample was placed into a tube containing 0.1 mm zirconium beads (Sigma-Aldrich Corp., St. Louis, MO) and agitated in a Mini-Beadbeater-9 for 2 min at the maximum settings. Samples were then incubated with MagNA Pure Bacterial Lysis Buffer, lysozyme, and lysostaphin as described above, without additional agitation in the Mini-Beadbeater-9. Proteinase K digestion and DNA purification were performed as described above.

### Quantitative PCR

NTM DNA extraction was quantified with qPCR targeting the *atpE* gene[24] under the following conditions: each 20 uL reaction contained 1X SYBR Green Master Mix, 500 nM of each primer, 0.75 mg/mL BSA, and 2 uL of undiluted sputum DNA. Cycling conditions were as follows: 95°C for 10 min x 1, then (95°C for 15 sec, 60°C x 1 min) for 40 cycles. Each run contained non-template control wells and a 10-fold dilution series of MABSC (ATCC 19977) genomic DNA. All samples were assayed in triplicate. The limit of detection for the qPCR reaction was 14 copies/reaction.

Differences in yield of total bacterial DNA between the lysis protocols were quantified with qPCR using primers targeting conserved regions of the bacterial 16S rRNA gene as described previously[11,25]. qPCR data were log-transformed and analyzed with paired t-tests. Data were analyzed with GraphPad Prism 6 (GraphPad Software, Inc., La Jolla, CA).

### DNA sequencing and sequence analyses

For the NTM culture-positive sputum and BAL samples, the V4 region of the 16S rRNA gene was amplified with barcoded primers and sequenced using the MiSeq Reagent Kit V2 (Illumina, San Diego, CA) and the MiSeq Illumina platform[14]. Raw sequencing data were processed with the mothur software package (version 1.35.0, downloaded on 5/21/15) as described in the MiSeq standard operating procedure [26]. OTU assignment, determination of presence or absence of NTM sequence reads, and relative abundance calculations were performed on sequence reads prior to subsampling. For calculations of alpha and beta diversity measures and Metastats[27] analysis, sequence reads were subsampled to 3,729, the smallest number of sequences obtained among the 30 samples. Sequencing data were analyzed with mothur and R[28].

## Results

### NTM detection by qPCR and 16S rRNA gene sequencing

**Fig 1:**
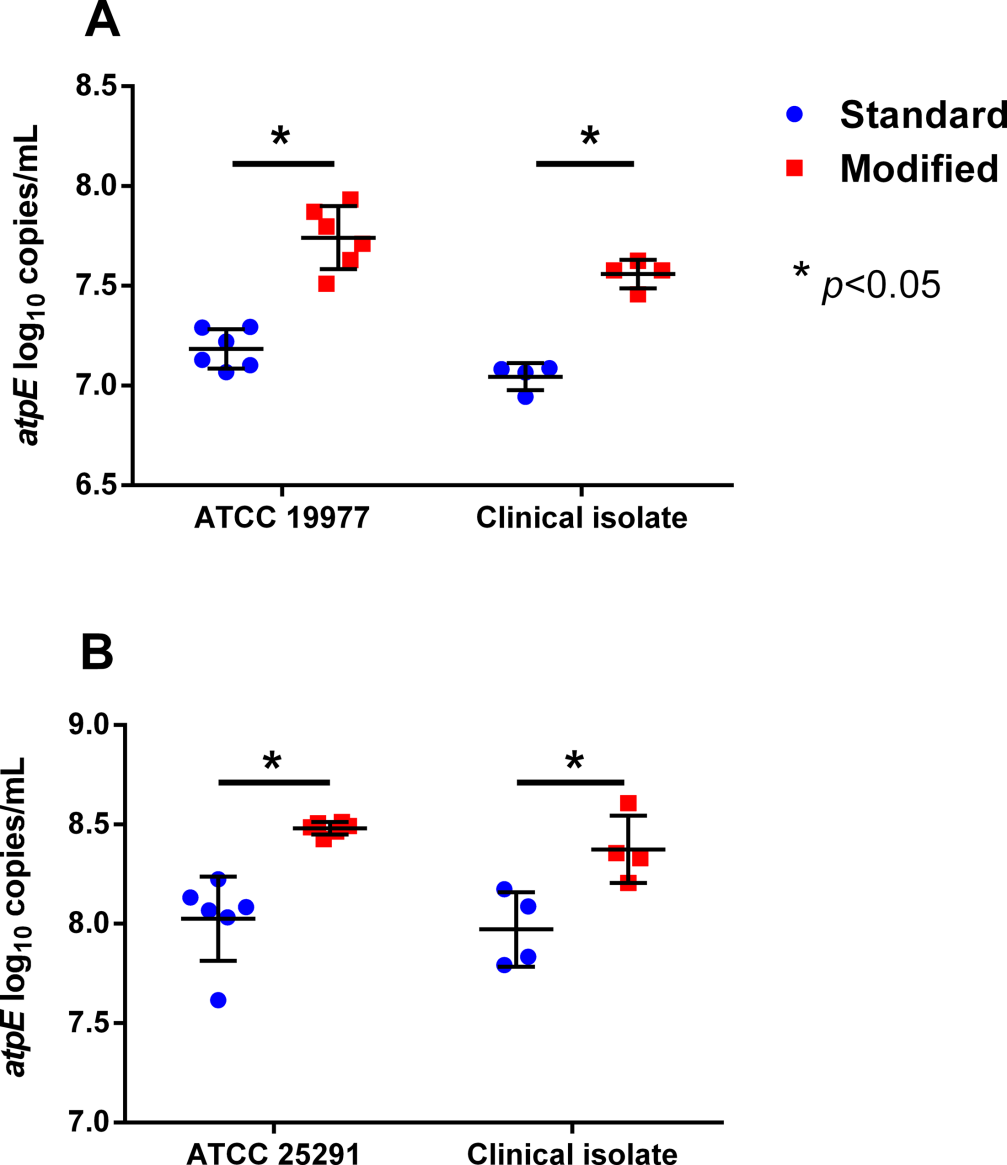
Improvement in NTM DNA extraction from spiked sputum samples with the modified as compared to the standard lysis protocol. Log_10_ *atpE* gene copies/mL in DNA extracted from sputum spiked with either (A) MABSC or (B) MAC using the standard (blue circles) or the modified (red squares) lysis protocols. Error bars indicate mean and SD.

Based on qPCR of the mycobacterial *atpE* gene, the use of the modified lysis protocol significantly increased the recovery of NTM DNA from the NTM-spiked sputum samples for each of the four NTM strains (Fig 1). The mean (SD) increases in log_10_ *atpE* gene copies/mL using the modified protocol compared to the standard protocol were 0.56 (0.19) for MABSC ATCC 19977 (p = 0.001, paired t-test), 0.51 (0.03) for the MABSC clinical isolate (p<0.001, paired t-test), 0.45 (0.23) for MAC ATCC 25291 (p = 0.005, paired t-test), and 0.4 (0.1) for the MAC clinical isolate (p = 0.004, paired t-test).

While NTM DNA was detected by qPCR targeting the *atpE* gene in three (20%) of the 15 culture-positive samples processed with the standard bacterial lysis protocol, six (40%) of the 15 samples were PCR-positive when processed with the modified lysis protocol (Table 1). All of the samples in which NTM DNA was detected by qPCR with either protocol were NTM smear-positive. NTM DNA was detected by qPCR in three (37.5%) of the eight smear-positive samples processed with the standard lysis protocol and in six (75%) of these samples processed with the modified lysis protocol. The qPCR-positive samples included both sputum and BAL samples, and samples that were culture-positive for either MABSC or MAC (Table 1).

**Table 1:** Characteristics of NTM culture-positive samples.

					NTM detection with *atpE* gene qPCR		NTM detection with 16S rRNA gene sequencing	
Sample number	Sample source	Appearance	NTM culture	Smear	Standard	Modified	Standard	Modified
1	Sputum	Thin	MABSC	-	-	-	-	-
2	Sputum	Thin	MAC	+	-	*-*	*-*	*-*
3	Sputum	Thin, bloody	MAC	+	-	+	-	+
4	Sputum	Thick	MABSC	-	-	-	-	-
5	Sputum	Thick	MAC	-	-	-	-	-
6	Sputum	Thick	MABSC	-	-	-	-	-
7	Sputum	Thick	MAC	-	-	-	-	-
8	BAL	Thin	MABSC	-	-	-	-	+
9	Sputum	Thin, bloody	MABSC	+	+	+	+	+
10	BAL	Thin	MAC	-	-	-	-	-
11	Sputum	Thick	MAC	+	+	+	-	+
12	Sputum	Thin	MAC	+	-	+	+	+
13	BAL	Thin	MAC	+	+	+	+	+
14	BAL	Thin	MABSC	+	-	-	-	+
15	Sputum	Thick	MABSC	+	-	+	+	+
Total					**3/15 (20%)**	**6/15 (40%)**	**4/15 (26.7%)**	**8/15 (53.3%)**

NTM 16S rRNA gene sequences were detected in four (27%) of the 15 culture-positive samples processed with the standard lysis protocol, and in eight (53.3%) of these 15 samples when processed with the modified lysis protocol (Table 1). NTM 16S rRNA gene sequences were not identified in any of the smear-negative samples processed with the standard lysis protocol, and in only one (14%) of these samples processed with the modified lysis protocol. NTM 16S rRNA gene sequences were detected in four (50%) of the eight smear-positive samples after processing with the standard lysis protocol, and in seven (87.5%) of these smear-positive samples after processing with modified lysis protocol. The samples that were positive for NTM 16S rRNA gene sequences included both sputum and BAL samples, samples with both thick and thin consistency, and samples that were culture-positive for either MABSC or MAC (Table 1).

**Fig 2:**
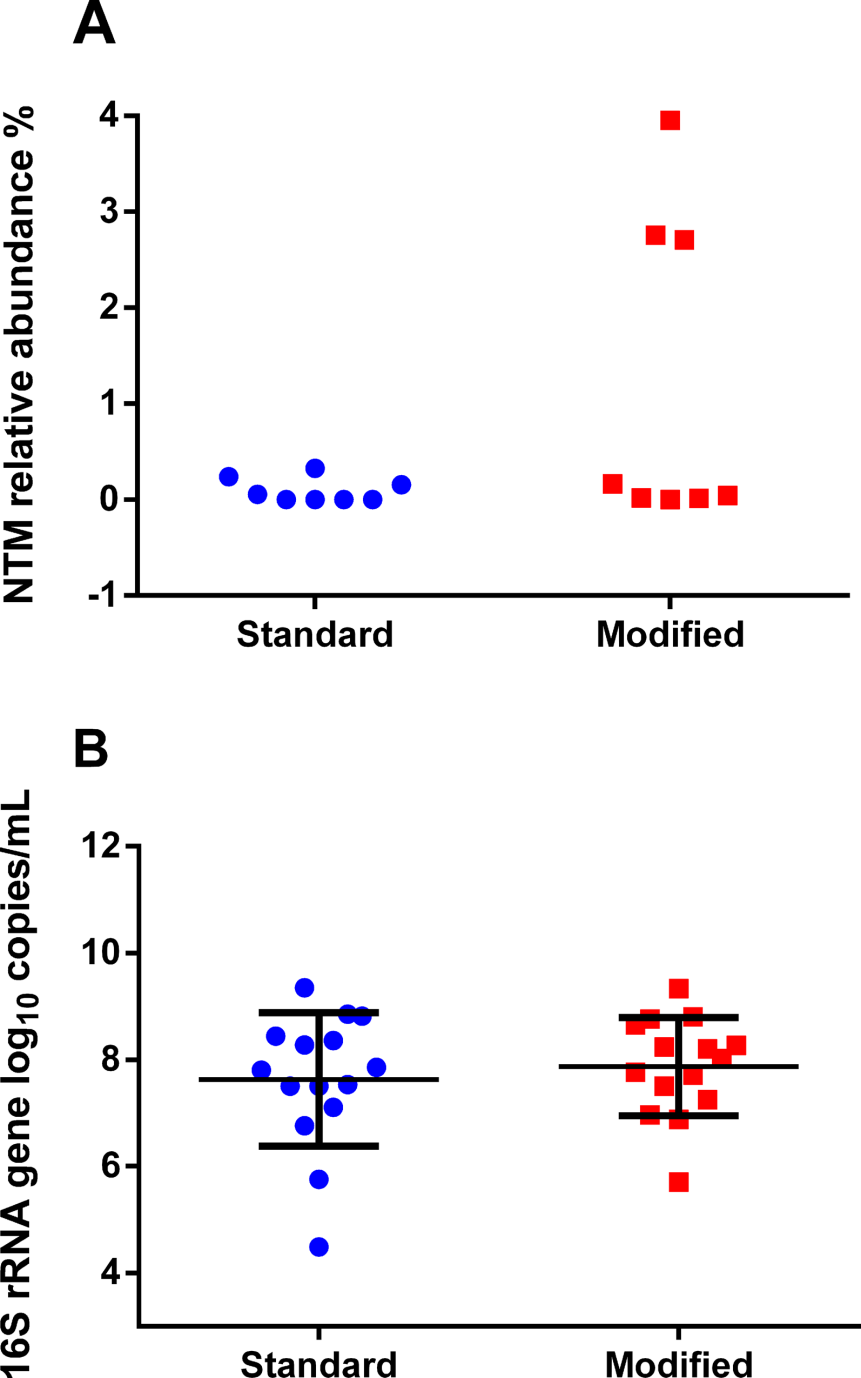
(A) Relative abundances of NTM OTUs and (B) total bacterial load in NTM culture-positive samples. (A) The mean relative abundance of NTM OTUs in the samples processed with the standard protocol was not significantly different from that observed when these samples were processed with the modified protocol (mean 0.098% and 1.21%, respectively; p = 0.08, paired t-test). (B) Total bacterial load in NTM culture-positive samples as measured by 16S rRNA gene qPCR did not significantly differ between lysis protocols. (p = 0.91, paired t-test). Error bars indicate mean and SD.

The relative abundances of NTM sequence reads in the eight samples in which NTM were detected by 16S rRNA gene sequencing after processing with the modified lysis protocol were compared to the relative abundances of NTM sequence reads detected in the same samples after processing with the standard lysis protocol. The mean relative abundance of NTM sequence reads increased from 0.098% (range 0.0%– 0.33%) with the standard lysis protocol to 1.21% (range 0.003% - 3.95%) with the modified lysis protocol; however, this difference was not statistically significant (p = 0.08, paired t-test) (Fig 2A). Based on qPCR targeting the bacterial 16S rRNA gene, total bacterial DNA did not differ between paired NTM culture-positive samples processed with the standard lysis protocol compared to the modified protocol (Fi 2B).

### Impact on community structure

**Fig 3:**
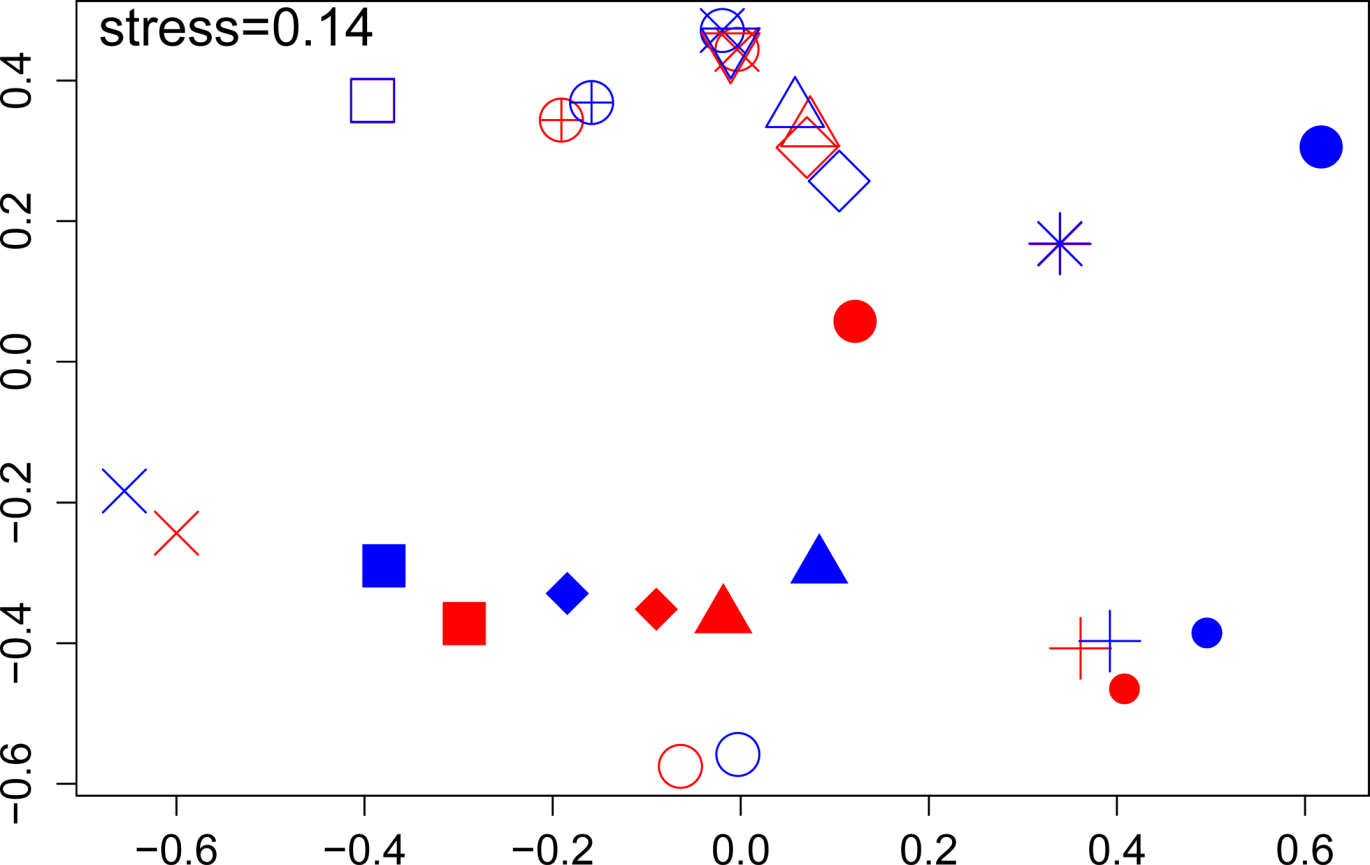
Impact of lysis method on community structure. Bray-Curtis-based nonmetric multidimensional scaling (NMDS) plot showing pairwise comparison of samples processed with the standard (blue symbols) or modified (red symbols) lysis protocols. Solid symbols represent paired samples with greater separation.

**Fig 4:**
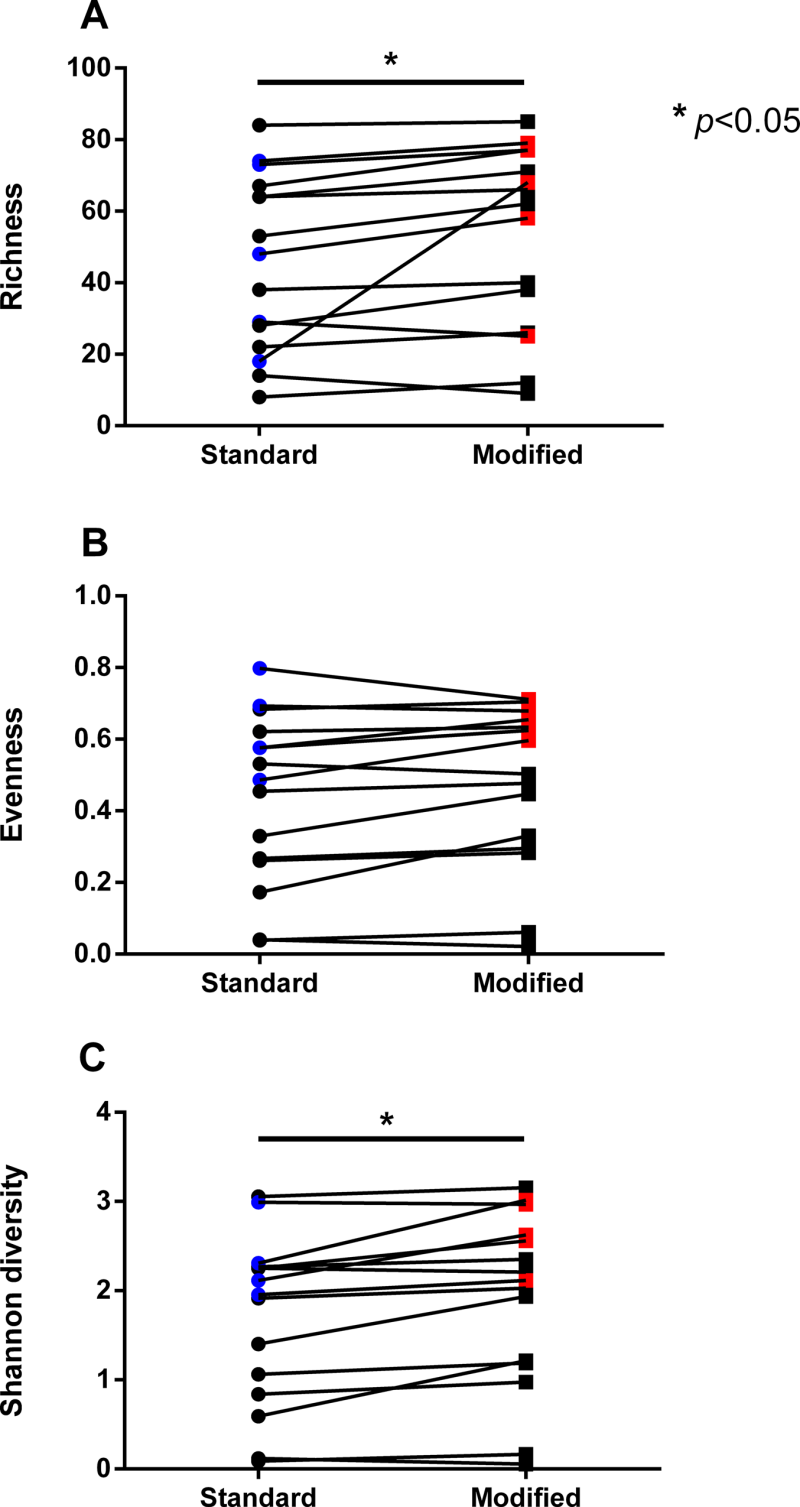
Differences in alpha diversity between lysis protocols. Samples processed with the modified protocol had higher levels of (A) richness (p = 0.04, paired t-test), and higher (C) Shannon diversity (p = 0.004, paired t-test) than samples processed with the standard protocol. (B) Evenness did not differ between the lysis protocols (p = 0.06, paired t-test). Blue and red symbols represent paired samples with greater separation on NMDS. Error bars indicate mean and SD.

The influence of the lysis protocols on bacterial community structure of the NTM culture-positive samples was assessed with a Bray-Curtis-based nonmetric multidimensional scaling (NMDS) plot (Fig 3). The majority of the sample pairs clustered closely together despite differences in lysis protocol. However, in five of the 15 sample pairs (solid symbols in Fig 3) some separation between samples was noted on the ordination plot. These 5 sample pairs tended to have higher levels of alpha diversity (higher Shannon diversity and evenness; blue and red symbols in Fig 4) than the 10 sample pairs that remained more tightly clustered despite differences in lysis protocol. Overall, the samples processed with the modified protocol had higher measures of richness and Shannon diversity than the samples processed with the standard protocol (Fig 4).

The potential for differential abundance of bacterial genera between the NTM culture-positive samples processed with the standard as compared to the modified lysis protocol was assessed. The ten most abundant OTUs in the sample set represented *Staphylococcus*, *Stenotrophomonas*, *Pseudomonas*, *Streptococcus*, *Prevotella*, *Veillonella*, *Haemophilus*, *Enterobacteriaceae*, *Firmicutes*, and *Actinomyces*; these did not significantly differ between the lysis protocols (Metastats[27] with Bonferroni correction for multiple comparisons).

## Discussion

With this study we illustrate the limitations of culture-independent detection of NTM from CF respiratory samples and demonstrate how alterations in the bacterial cell lysis method can be employed to improve NTM sequence-based detection. Despite the significant improvements seen in NTM DNA extraction with the modified lysis protocol, NTM sequences were detected by 16S rRNA gene sequencing in only about half (53%) of the NTM culture-positive samples (87% of smear-positive and 14% of smear-negative samples) included in our study. This decreased sensitivity of 16S rRNA gene sequencing for NTM as compared to culture are consistent with findings of MTb pulmonary infection in a non-CF population, in which *Mycobacterium* was identified by 16S rRNA gene sequencing in only 39% (29/75) of sputum samples from MTb-infected individuals[10]. Consistent with a recent study in which qPCR was less sensitive than 16S rRNA gene sequencing for detection of *Staphylococcus aureus* from CF respiratory samples[29], qPCR of the *atpE* gene in our study was less sensitive than 16S rRNA gene sequencing in detecting NTM in CF respiratory samples.

The improvement in NTM DNA sequence detection with use of the modified lysis protocol supports inadequate bacterial lysis as a contributing factor to the underrepresentation of NTM in culture-independent studies of the CF microbiome. NTM are notoriously difficult to lyse due to the layer of glycopeptidolipids and mycolic acid that make up the NTM cell wall[30]. Previous work by others has shown that NTM lysis and DNA yields can be improved by enhancing the physical disruption of mycobacterial cell walls[15,18,19,21]. We similarly observed that the greatest increase in NTM DNA yield from CF respiratory samples was obtained by altering bead beating conditions. More specifically, the changes from glass beads to the denser zirconium beads and decreasing the sample volume as described in our modified protocol provided the greatest increase in NTM yield, with a minimal impact on the 16S rRNA gene sequence based measures of bacterial community structure in the majority of the NTM culture-positive respiratory samples.

These and other efforts to optimize NTM DNA extraction have focused on cultured NTM isolates[15,16,18,21], or on respiratory samples from individuals with MTb infection[31]. We are not aware of previous studies to enhance NTM DNA extraction from CF respiratory samples. CF sputum is a complex biologic matrix dominated by neutrophil-derived DNA polymers and F-actin that differs from the respiratory secretions found in other chronic pulmonary processes such as asthma or bronchitis[32]. We reiterate that while our modified cell lysis protocol increased NTM yield from CF respiratory samples, other approaches may be more appropriate for other biological or environmental sample types.

In addition to the difficulty of lysing NTM bacterial cells, other factors likely also contribute to the poor performance of culture-independent approaches in identifying NTM in CF respiratory samples. MABSC and MAC, the primary NTM species found in CF infections, have genomes that harbor single 16S rRNA gene operons[33,34]. This is in contrast to other bacterial genera, which typically have genomes that include multiple copies of this operon[35]. The genome of *Pseudomonas aeruginosa*, for example, includes four copies of the 16S rRNA gene operon[35]. NTM infection also often may involve lower levels of bacterial burden than infection with other known CF pathogens. For instance, NTM smear positivity is observed with a bacterial density of ≥ 10^3^ cfu/mL[36]. Smear positivity is typically thought to indicate a high burden of infection[37], but occurs in the minority of individuals with CF and NTM pulmonary infection[1]. This is in contrast to infection with *P*. *aeruginosa*, where bacterial density in the CF airways can often exceed ~10^7^ cfu/mL[38]. The difficulty in lysing NTM bacterial cells, the single 16S rRNA gene operon, and the low density of NTM all likely contributed to the lack of culture-independent NTM detection in the smear-negative samples despite the lysis protocol modifications.

We identified NTM sequences by 16S rRNA gene sequencing in 27% of the NTM culture-positive samples processed with the standard protocol. This is in contrast to the absence of NTM sequences found in the 16 culture-positive sputum samples that were analyzed by next-generation sequencing of the 16S rRNA gene in our prior studies[7,11–14]. We note, however, that in these previous studies, DNA sequencing was performed using the Roche 454 sequencing platform. We suspect that the greater sequencing depth of the MiSeq Illumina sequencing platform used in the current study may account for the detection of NTM sequences in the small number of samples processed with the standard protocol. We recognize that multiple variables, or combinations of variables, may influence DNA sequence-based detection of NTM. In addition to bacterial lysis, DNA extraction, and sequencing platform, these variables also include the variable region of the 16S rRNA gene targeted and the analytic pipeline used for 16S rRNA sequence analysis[39].

In this study, we limited our testing of the lysis protocols to sputum and BAL samples containing MABSC and MAC, as these are the NTM species most commonly isolated from the CF airways[1,2]. The cell wall mycolic acid composition varies between NTM species in ways that influence susceptibility to lysis methods[16,21]. Although our findings were consistent across the four NTM strains tested, and across multiple CF clinical samples, we cannot confidently extrapolate our results to all other NTM species.

Understanding the limitations of DNA sequence-based detection of NTM from CF respiratory samples is a necessary first step for future culture-independent studies of the microbial ecology of NTM infection in the CF. Identifying CF airway microbial community structures (e.g., members of the communities and their distribution) that relate to NTM acquisition, NTM pulmonary disease, and/or treatment response, will be critical steps in identifying clinically useful biomarkers of disease and in understanding the pathophysiology of NTM disease in CF.
